# Benzophenone-3 remodels gut microbiota diet-dependently to exacerbate non-alcoholic fatty liver disease in zebrafish

**DOI:** 10.3389/fmicb.2025.1694753

**Published:** 2025-10-24

**Authors:** Junyan Tao, Linxuan Tian, Qinyuan Yang, Yao Jiang, Yubo Liu, Junli Wang, Xiong Chen, Hui Gao

**Affiliations:** ^1^The Key Laboratory of Environmental Pollution Monitoring and Disease Control, Ministry of Education, School of Public Health, Guizhou Medical University, Guiyang, Guizhou, China; ^2^Department of Forensic Medicine, Guizhou Medical University, Guiyang, Guizhou, China; ^3^Key Laboratory of Fertility Preservation and Maintenance of Ministry of Education, School of Basic Medicine, Ningxia Medical University, Yinchuan, China

**Keywords:** Benzophenone-3, diet-dependent manner, gut microbiota, NAFLD, zebrafish

## Abstract

Benzophenone-3 (BP3), a prevalent organic UV filter found in aquatic environments and human tissues, poses potential metabolic risks. This study investigated the combined effects of BP3 (10 μg/L) and a high-fat diet (HFD, 24% crude fat) on non-alcoholic fatty liver disease (NAFLD) development in zebrafish, focusing on gut-liver axis disruption via microbiota. Co-exposure to BP3 and HFD significantly worsened hepatic steatosis, as evidenced by increased triglyceride levels, lipid droplets accumulation, and oxidative damage (elevated hepatic MDA levels and decreased hepatic CAT activity). Additionally, this combined exposure induced gut dysbiosis characterized by a marked decrease in *Bacteroidota* and *Fusobacteriota*, along with increased proportions of *Proteobacteria* and *Actinobacteriota*, and an altered *Firmicutes/Bacteroidota* ratio. This dysbiosis compromised intestinal barrier integrity, leading to anterior/middle intestines villus atrophy, endotoxin translocation, and hepatic inflammatory activation. Notably, BP3 demonstrated a diet-dependent effects, depleting *Bacteroidia* under normal diet while increasing *Gammaproteobacteria* under HFD. These findings, highlight that BP3 synergizes with HFD to disrupt the microbiota-gut-liver axis, accelerating NAFLD progression, and emphasize the host’s metabolic status as a critical determinant of pollutant-microbiota interactions and toxicity. This diet-dependent effect challenges isolated toxins risk assessments, underscoring the need to incorporate dietary context into NAFLD prevention and environmental health rules.

## 1 Introduction

Benzophenone-3 (BP3), an organic UV filter extensively used in sunscreens and personal care items, is commonly found in aquatic environments due to its poor degradability and tendency to accumulate in lipid-rich tissues (Fent et al., 2010; Kim and Choi, 2014). Consistent with this environmental persistence, studies have showed that BP3 concentration spanning from ng/L to mg/L, with the most elevated levels found in surface water samples (43–44 μg/L) and wastewater (3.97 mg/L) in the UK (Kasprzyk-Hordern et al., 2009; Kim and Choi, 2014; Ramos et al., 2015, 2016). Human exposure to BP3 is widespread, as evidenced by its detection in 49 out of 50 commercial sunscreen products sampled in the United States in 2021 (Li and Kannan, 2022). Following dermal application, plasma levels concentrations of BP3 can peak at 200–300 μg/L, and lactating women have demonstrated detectable serum BP3 levels ranging from 1.4 to 59.2 μg/L (Hines et al., 2015; Janjua et al., 2008; Tarazona et al., 2013). The frequent presence of BP3 in aquatic environments and human tissues raises concerns regarding its potential health risks to both aquatic organisms and humans. Previous research has mainly focused on the endocrine effects of BP3, due to its structural resemblance to estradiol (Xu et al., 2021). However, our prior studies identified BP3-induced neurotoxicity in embryonic and adult zebrafish at a concentration of 10 μg/L (Bai et al., 2023; Tao et al., 2020). Recent studies have additionally indicated that environmentally relevant concentrations of BP3 can induce hepatic oxidative stress and gut dysbiosis in fish (Wang et al., 2024; Zhang et al., 2020), highlighting significant metabolic risks of low-dose exposure. Considering the frequent incidence of metabolic disorders arising from environmental and dietary factors, along with BP3’s ability to disrupt liver and gut functions, it is crucial to explore the potential interaction between BP3 and dietary components, particularly high-fat diets, in worsening conditions.

Non-alcoholic fatty liver disease (NAFLD) represents a significant global public health issue, with prevalence rates exceeding 30% in various regions (Middle East: 37.79%, South America: 30.45%, Asia: 27.37%) (Younossi et al., 2016). It is anticipated that NAFLD-related mortality will surge by 178% by 2030, driven by widespread high-fat diets (HFD) consumption (Estes et al., 2018). The primary pathological mechanisms involve oxidative stress, stemming from the excessive of reactive oxygen species due to the inhibition of peroxisome proliferator-activated receptor alpha (PPARα), and disruption of the gut-liver axis, leading to lipopolysaccharide (LPS) translocation and toll-like receptor 4 (TLR4)/nuclear factor kappa B (NF-κB) pathway activation (Chen et al., 2020; Li et al., 2021). These mechanisms collectively facilitate the progression from simple fatty liver to steatohepatitis. Notably, the gut-liver axis is significantly involved in the advancement of NAFLD. Gut microbiota dysbiosis compromises the intestinal barrier integrity, facilitating endotoxin (e.g., LPS) translocation. This translocation triggers hepatic inflammation via TLR4 (Miura and Ohnishi, 2014). Furthermore, bile acid dysregulation worsens this process, enhancing lipid accumulation and establishing harmful cycles with other liver diseases (Tripathi et al., 2018). Importantly, environmental pollutants are increasingly acknowledged for their significant contribution to NAFLD exacerbation by amplifying these core mechanisms. For example, Bisphenol A (BPA) accumulates in livers impaired by NAFLD due to reduced glucuronidation capacity, and further exacerbates NAFLD by promoting lipid synthesis, oxidative stress, and insulin resistance, forming a vicious cycle (Verstraete et al., 2018). Similarly, PM2.5 disrupts hepatic metabolic balance by inhibiting the PPARα pathway, leading to lipid accumulation, impaired lipid export, and subsequent inflammation that worsens NAFLD progression (Zheng et al., 2013). Nevertheless, knowledge gaps persist concerning emerging UV filters like BP3, particular regarding their potential to disrupt lipid metabolism, their synergistic effects with HFD in accelerating NAFLD progression, gut microbiota-mediated within the gut-liver axis and how host’s metabolic status (e.g., HFD) modulates BP3-microbiota crosstalk to drive metabolic outcomes.

The zebrafish (*Danio rerio*) has become a pivotal supplementary model to mammalian systems, owing to its transparent embryonic development, high fecundity, and 70% genomic homology with humans (Chang et al., 2023; Li et al., 2024). Despite limited research on fish, particularly adult specimens, their endocrine system functionality and identified metabolites resemble those of terrestrial vertebrates (Grinberg et al., 2023). The hepatic architecture of zebrafish encompasses evolutionarily conserved functional elements like hepatocytes and cholangiocytes, while its highly conserved xenobiotic metabolism pathways enhance its utility in liver disease research (Chang et al., 2023; Li et al., 2024). Dietary interventions have established various NAFLD zebrafish models. For instance, a high-fat diet with a 24% fat content can induce NAFLD within 2 weeks (Guo et al., 2021). Therefore, leveraging its conserved hepatic architecture and metabolic pathways, along with efficient dietary induction strategies, the zebrafish model offers a robust platform for studying the role of environmental pollutants in NAFLD pathogenesis, exploring pollutant-microbiota-host interactions under different metabolic states and exploring potential therapeutic interventions.

In this study, adult zebrafish were utilized to explore the synergistic effects of BP3 with high-fat diets on NAFLD susceptibility and to elucidate the underlying mechanisms, with a specific focus on the role of the gut-liver axis. Crucially, we aimed to investigate how the host’s metabolic background (normal vs. high-fat diet) influences the impact of BP3 on gut microbiota and the subsequent cascade leading to NAFLD exacerbation. We hypothesized that the co-exposure to BP3 and a high-fat diet would synergistically exacerbate NAFLD progression. We proposed that this effect is mediated through a diet-dependent remodeling of the gut microbiota, which subsequently leads to intestinal barrier dysfunction, endotoxin translocation, and hepatic inflammation. The evaluation involves examining hepatic lipid accumulation, liver inflammation-related gene expression, metabolism-related genes associated with liver steatosis, and levels of hepatic oxidative stress as characteristic features of NAFLD. Additionally, we explored the role of the gut-liver axis in the synergistic toxic mechanism by conducting a comprehensive analysis of intestinal oxidative stress, intestinal mechanical barrier integrity, serum levels of intestinal-derived LPS, and the composition and diversity of gut microbiota. This study delves into the effects of environmentally relevant concentrations of BP3 in combination with high-fat diets on the development of NAFLD in zebrafish, aiming to provide mechanistic insights into how combined environmental and dietary risk factors drive NAFLD pathogenesis.

## 2 Materials and methods

### 2.1 Chemical stock solutions

Benzophenone-3 (CAS#131-57-7) with a purity exceeding 98% was acquired from Sigma, and subsequently solubilized in dimethyl sulfoxide (DMSO) to form a concentration of 100 mg/L. The stock solution was stored in a cool, dark environment at −20 °C for subsequent dilutions. Prior to the experiment, the BP3 stock solution underwent a 10,000-fold dilution to produce the working solution. Specifically, 700 μL of the BP3 stock solution was added to 7 L of breeding water in a 8 L tank, with a consistent DMSO concentration of 0.01% maintained across all experimental groups.

### 2.2 Zebrafish husbandry and experimental design

Adult zebrafish were cultured in a semi-static environment at 28 °C with a light/dark cycle of 14/10 h, following established zebrafish breeding procedures (Westerfield, 2000). The system received reverse osmosis-filtered water with added sea salt to achieve a conductivity level of 500–1000 μS/cm. Zebrafish aged 4 months old were randomly allocated into four distinct experimental groups (*n* = 3 replicates, with 28 fish per replicate for each group): ND (normal diet with 6% crude fat), ND + BP3 (normal diet with 6% crude fat) + 10 μg/L BP3, HFD (high fat diet with 24% crude fat), and HFD + BP3 (high fat diet with 24% crude fat) + 10 μg/L BP3. All subsequent procedures and data collection were performed by investigators blinded to the group assignments. Each group was housed in tanks with 8 L breeding water, and the diets, sourced from Trophic Animal Feed High-Tech Co., Ltd., (Nantong, China), were fed twice daily at 25 mg/meal (approximately 8% body weight). The exposure solution was changed daily, with BP3 concentration maintained at 10 μg/L, a concentration deemed environmentally relevant in line with previous studies (Kim and Choi, 2014; Ramos et al., 2015; Tao et al., 2020; Tian et al., 2025). Following a 7-days exposure period, the zebrafish were anesthetized in an ice-water bath until opercular movement ceased. The fish were subsequently dried on filter paper, and weighted to determine their total body weight. Following aseptic conditions, the abdominal cavity was opened with dissecting forceps to expose the internal organs. The liver and intestine were then carefully excised, being careful to avoid neighboring tissues, and immediately transferred to a balance for organ weight measurement. A portion of the liver and intestines was fixed in 4% paraformaldehyde (PFA, Cat. No. P1110, Solarbio, China) for subsequent histological examination (*n* = 3 replicates, with 6 fish per replicate for each group). The remaining liver and intestinal tissues, along with serum, were immediately stored at −80 °C for further analyses (*n* = 3 replicates, with 22 fish per replicate for each group). Additionally, the hepatosomatic index (HSI) and Intestosomatic index (ISI) values were also calculated (Abasubong et al., 2023). Zebrafish procedures were conducted in compliance with the guidelines of Institutional Animal Care and Use Committee at Guizhou Medical University. The experimental protocol received approval under permit number 2100215.

### 2.3 Quantifying the bioaccumulation of BP3 contents

Analysis of BP3 content in zebrafish liver and intestinal tissues was conducted using liquid chromatography-mass spectrometry (LC-MS) following our previous established protocols (Tian et al., 2025). In short, livers or intestines tissues (*n* = 3 replicates, with 4 livers/intestines per replicate for each group) were homogenized in methanol: water (1:3), ultrasonicated for 15 min, and incubated at 4 °C for 1 h. After centrifugation (12,000 *g*, 20 min, 4 °C), supernatants were dried under N_2_, reconstituted in methanol: water (7:3), and re-centrifuged (12,000 *g*, 10 min, 4 °C). The extracts were analyzed by LC-MS (AB 4500QTRAP, SCIEX, USA) employing a Titank-F5 column (2.1 mm × 100 mm, 1.8 μm; Guangzhou FLM Scientific Instruments Co., Ltd., China) with acetonitrile: water (50: 50, 1 mM HOAc) mobile phase at a flow rate to 0.4 mL/min. Quantitation of BP3 employed positive ion mode (Q1/Q3: 229.0/151.0 or 105.0). A representative chromatogram of BP3 analyzed under the described LC-MS conditions can be found in the Supplementary Figure 1.

### 2.4 Histopathological examination

Following fixation in 4% PFA for a period of 48 h, the excised livers and intestines [*n* = 3 replicates, with 3 livers/intestines (anterior, middle and posterior) per replicate for each group] underwent dehydration in an ethanol gradient and clarification with xylene. The tissues were then embedded in molten paraffin for sectioning. Sections of 4 μm thickness were obtained utilizing a HistoCore Multicut microtome (Leica Microsystems, Wetzlar, Germany). Subsequently, the sections underwent deparaffinization in xylene, rehydration in ethanol, staining with hematoxylin for 1 min, differentiation with 0.1% hydrochloric acid ethanol, rinsing in distilled water, and counterstaining with eosin (Cat. No. G1120, Solarbio, China) for 30 s. Following stain removal, sections were mounted with neutral resin. Microscopic examination of the liver and intestine structure was conducted using a NanoZoomer S60 digital slide scanner (Hamamatsu, Japan).

### 2.5 Oil Red O staining

The liver samples (*n* = 3 replicates, with 3 livers per replicate for each group) underwent fixation overnight in PFA at 4 °C, followed by dehydration in a 30% sucrose solution. Subsequently, they were embedded in Tissue-Tek O.C.T compound and preserved for at −80 °C until sectioning. Transverse sections of 12 μm were prepared from the liver tissue using a freezing microtome (Leica, Germany). Upon reaching room temperature, the sections were fixed in 4% PFA for 10 min, and then treated with Oil Red O solution (Cat. No. G1260, Solarbio, China) in the absence of light. Subsequently, the sections were differentiated with 60% isopropyl alcohol, counterstained with hematoxylin for 1 min, washed to remove excess stain, and mounted with glycerin gelatin. Lipid deposition in liver cells of zebrafish from each group was visualized microscopically.

### 2.6 Hepatic lipids levels in zebrafish liver

Triglyceride (TG) and free fattyacid (FFA) levels in livers (*n* = 3 replicates, with 4 livers per replicate for each group) were quantified using the corresponding commercial kits (Solarbio, China) following the manufacturer’s protocols. Briefly, the samples were homogenized in extraction solution on ice, then centrifuged at 8000 *g* for 10 min at 4 °C. The resulting supernatant was mixed with the corresponding color-developing reagents for a colorimetric reaction. Absorption values were determined at 420 nm for TG and 550 nm for FFA using a microplate reader (Multiskan GO, Thermo Scientific, America).

### 2.7 LPS levels in zebrafish serum

Lipopolysaccharide (LPS) levels in zebrafish serum were measured using ELISA kits (JL13861, Jonln Biotechnology) following the manufacturer’s instructions. Blood samples were collected from eight zebrafish per experimental group by caudal amputation following anesthesia on ice (*n* = 3 replicates, with pooled whole blood from 8 fish per replicate for each group) (Pedroso et al., 2012). Pooled whole blood from each group was centrifuged at 1,000 *g* for 20 min at 4 °C to separate serum, which was then transferred to sterile tubes. Serum samples were incubated with HRP-conjugated antibodies in microplate wells at 37 °C for 1 h. After washing, chromogenic reagent was added and incubated at 37 °C for 15 min. The reactions were terminated with stopping solution, and the absorbance at 450 nm was measured.

### 2.8 Analysis of redox biochemical indicators

Malondialdehyde (MDA) and catalase (CAT) levels in zebrafish livers and intestines were assessed using the corresponding commercial kits A003-1-2 and A007-1-1 (Nanjing, China), following the manufacturer’s methods. Briefly, the sample from four individual zebrafish in the same group were pooled to create one replicate (*n* = 3 replicates, with 4 livers per replicate for each group). The samples were homogenized in extraction solution on ice, then centrifuged at 8000 *g* for 10 min at 4 °C. The supernatant was mixed with the corresponding reagent for the reaction. Absorption values for MDA and CAT were determined at 532 and 405 nm, respectively, employing a microplate spectrophotometer (Thermo Scientific, America).

### 2.9 RNA extraction and quantitative real-time PCR analysis

Following exposure, liver tissues from three zebrafish were pooled per biological replicate, homogenized in TRIzol reagent (Life Technology, USA), and total RNA was quantified using the microplate spectrophotometer. Only RNA samples with OD260/OD280 ratios of 1.8–2.0 were retained for next processing. Subsequently, 3,000 ng of total RNA was reverse-transcribed in 10 μL reaction with the RevertAid First Strand cDNA Synthesis Kit (Thermo Fisher Scientific, USA), containing 0.5 μL oligo (dT) 18, 2 μL 5 × Reaction buffer, 0.5 μL RiboLock RNase inhibitor (20 U μL^1^), 1 μL 10 mM dNTP mix, and 0.5 μL Revertaid M-MuLV reverse transcriptase (200 U μL^1^), and nuclease-free water to adjust the final volume. Quantitative PCR (qPCR) was then performed in 10 μL volumes containing 5 μL TB Green Premix Ex Taq II (Takara, Japan), 0.8 μL each of forward/reverse primers (10 μM), 0.8 μL cDNA template, and 3.4 μL nuclease-free water, amplified on a CFX96 Real-Time PCR System (Bio-Rad) under thermal cycling conditions described previously (Sun et al., 2023). Gene expression was analyzed via the 2^ΔΔ*CT*^ method normalized to β-actin, with all primers (Supplementary Table 1) synthesized by Shenggong Biotech (Shanghai, China).

### 2.10 Microbiome analysis of intestines

After exposure, fish underwent a 24-h fast and their entire intestines were aseptically isolated following the previous protocol (Milligan-McClellan et al., 2011). The intestinal surface was subsequently washed with sterile PBS. Three intact intestinal segments were combined to form one replicate (*n* = 3, with 3 intestines per replicate for each group), which was immediately snap-frozen and stored at −80 °C for further analysis.

Microbial genomic DNA was extracted using the FastPure Stool DNA Isolation Kit (MJYH, Shanghai, China). The V3-V4 region of the bacterial 16S rRNA gene was amplified with primers 338F (5′-ACT CCT ACG GGGA GCA CAG-3′) and 806R (5′-GGA CTA CHV GGG TWT CTA AT-3′). Amplified products were purified, quantified, and sequenced on the Illumina NextSeq 2000 PE300 platform (San Diego, USA). Paired-end reads were merged using FLASH (v0.19.6)^1^, and OTU clustering at 97% similarity. Alpha diversity indices were computer using mothur (v1.30.2)^2^, while beta diversity was assessed via Principal Component Analysis (PCA) to evaluate structural similarities in microbial communities across samples. Differential microbial taxa across groups were identified by Linear Discriminant Analysis Effect Size (LEfSe)^3^, with results visualized on the Majorbio Cloud Platform^4^.

### 2.11 Statistical analysis

In our study, zebrafish were randomly assigned to distinct experimental groups (control, BP3, HFD, and BP3 + HFD) before treatment initiation. Initially, zebrafish of identical age and weight were grouped together and then randomly allocated to specific groups using numbered tags placed in a container. This method ensured an equitable distribution of baseline characteristics (e.g., age, weight, activity level) among all groups, thereby reducing the potential for selection bias associated with subjective grouping. Data are presented as mean ± SEM using GraphPad Prism 9.5 and SPSS 26.0 under a double-blind design, where individuals involved in BP3/diets preparation were isolated from those conducting exposures and measuring endpoints, all of whom were blinded to group assignment. Normality and homogeneity of variance were evaluated via the Kolmogorov-Smirnov (K-S) test (normality criterion: *P* > 0.10) and Levene’s test (homoscedasticity criterion: *P* > 0.05), respectively. Multiple-group comparisons employed one-way ANOVA; upon detecting significant differences (*P* < 0.05), pairwise analyses used Bonferroni test correction. Additionally, five predefined pairwise comparisons (ND + BP3 vs. ND; HFD vs. ND; HFD + BP3 vs. HFD; HFD + BP3 vs. BP3; HFD + BP3 vs. ND) were examined using Welch’s-corrected independent *t*-tests (parametric) or Mann-Whitney U tests (non-parametric). Monotonic variable relationships were assessed by Spearman’s rank correlation, with *P* < 0.05 considered statistically significant throughout.

## 3 Results

### 3.1 Quantification of BP3 in liver and intestine

**FIGURE 1:**
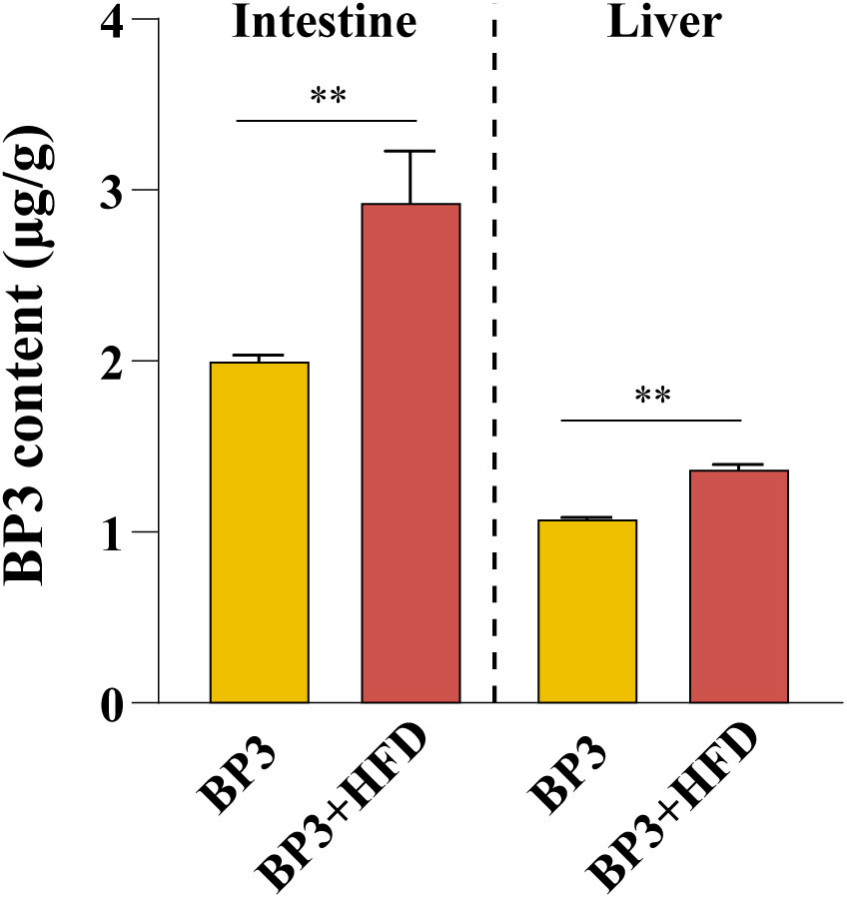
Bioconcentrations of BP3 in the intestine and liver of zebrafish. Values plotted are mean ± SEM. **P < 0.01 indicate significant differences when compared with BP3 group.

The BP3 levels were significantly higher in the intestines of the BP3 + HFD group compared to the BP3 group (*P* = 0.006). Similarly, BP3 levels in the liver exhibited a comparable increase (*P* = 0.003), with liver content being lower than that in the intestine. BP3 levels in the control group were below the limit of detection (LOD) (Figure 1).

### 3.2 Organ biometric parameters in zebrafish

**FIGURE 2:**
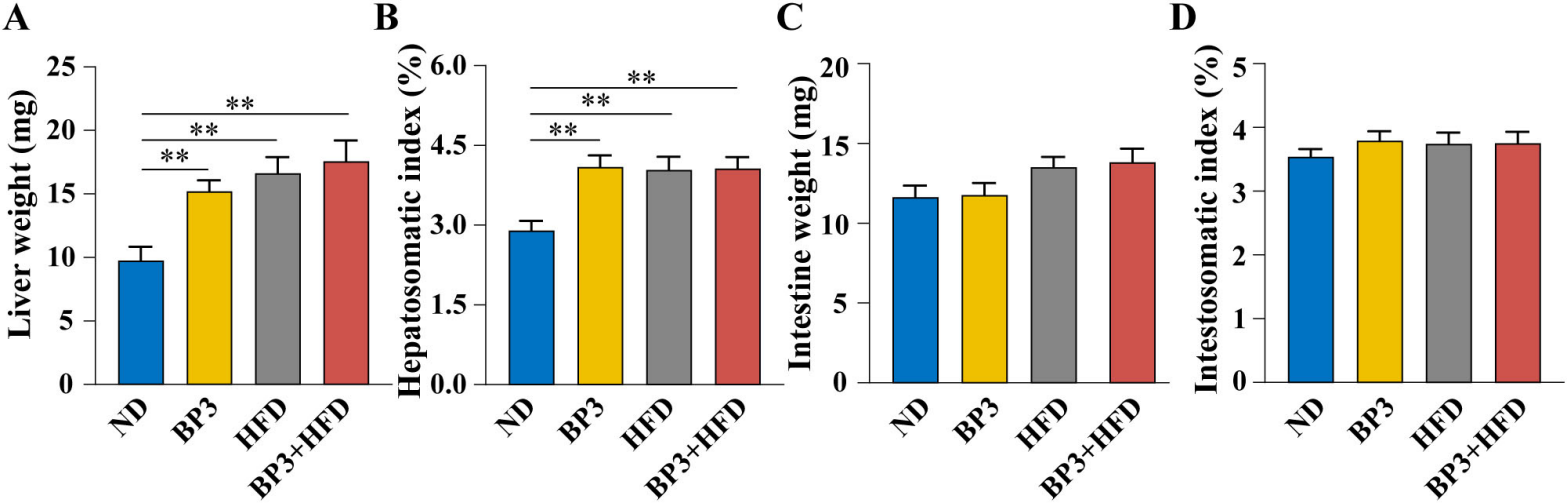
Effects of BP3 exposure on growth parameters in zebrafish under ND and HFD conditions. **(A)** Liver weight, **(B)** hepatosomatic index (HSI = liver weight × 100/body weight), **(C)** intestine weight, **(D)** intestosomatic index (ISI = intestine weigh × 100/body weight). Values plotted are mean ± SEM. **P < 0.01 indicates significant differences between the control and exposure groups.

All treatment groups exhibited a significant increase in liver weight (*P* = 0.007 for BP3, *P* = 0.004 for HFD, *P* = 0.001 for BP3 + HFD) and hepatosomatic index (*P* = 0.002 for BP3, *P* = 0.002 for HFD, *P* = 0.001 for BP3 + HFD) compared to the ND group (Figures 2A, B). Conversely, there were no statistically significant alterations in intestinal weight and intestosomatic index (*P* > 0.05) (Figures 2C, D).

### 3.3 Hepatic lipid accumulation and oxidative damage in zebrafish

**FIGURE 3:**
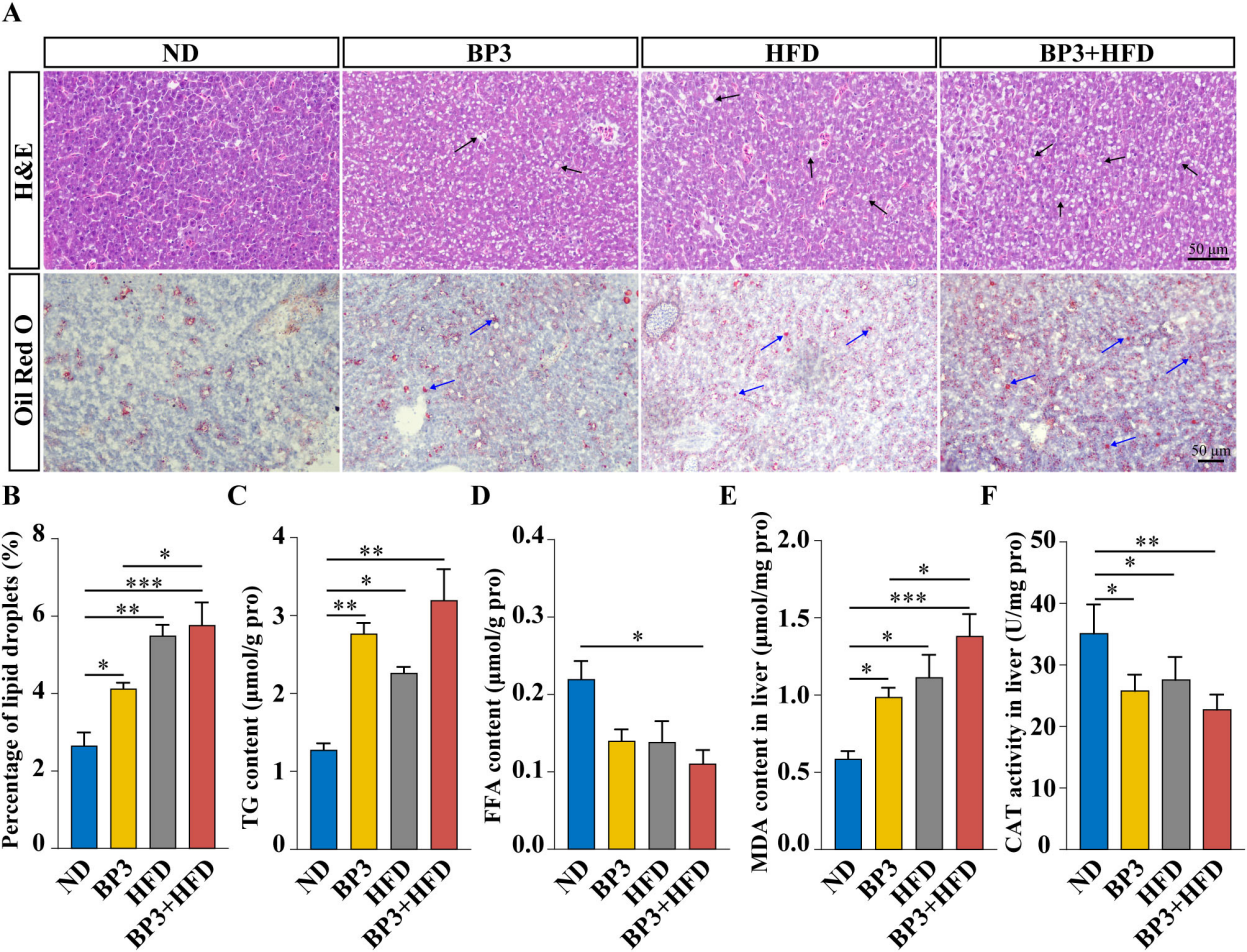
Effects of BP3 exposure on hepatic steatosis and oxidative damage in zebrafish under ND and HFD conditions. **(A)** H&E staining (magnification at 400× and scale bar: 50 μm) and Oil Red O staining (magnification at 200× and scale bar: 50 μm) of representative liver sections belonging to different groups. The black arrows indicate lipid vacuoles, and the blue arrows indicate lipid droplets. **(B)** Semi-quantified area of lipid droplets. **(C)** Hepatic triglyceride concentrations. **(D)** Hepatic free fatty acids concentrations. **(E)** MDA contents and **(F)** CAT contents in liver. Values plotted are mean ± SEM. *P < 0.05, **P < 0.01, ***P < 0.001 indicate significant differences between the control and exposure groups.

Hepatocellular ballooning and lipid droplet deposition were observed in all treatment groups compared to the ND group (Figure 3A), as showed by HE and Oil Red O staining. Semi-quantitative assessment confirmed a significant increase in lipid droplet accumulation in all treatment groups compared to the ND group (*P* = 0.043 for BP3, *P* = 0.001 for HFD, *P* < 0.001 for BP3 + HFD). Notably, the BP3 + HFD group exhibited a significant higher lipid droplet content than the BP3 group (*P* = 0.024) (Figure 3B). Biochemical analysis demonstrated a significant elevation in TG levels (*P* = 0.006 for BP3, *P* = 0.029 for HFD, *P* = 0.003 for BP3 + HFD) (Figure 3C), and a decrease in FFA content (*P* = 0.022 for BP3 + HFD) (Figure 3D). Furthermore, hepatic oxidative damage markers displayed marked dysregulation, with the lipid peroxidation marker MDA content significantly increased (*P* = 0.044 for BP3, *P* = 0.014 for HFD, *P* < 0.001 for BP3 + HFD). Moreover, MDA content in the BP3 + HFD group were significantly higher than those in the BP3 group (*P* = 0.047) (Figure 3E). Meanwhile, the antioxidant enzyme CAT activity was significantly decreased in all treatment groups (*P* = 0.013 for BP3, *P* = 0.044 for HFD, *P* = 0.002 for BP3 + HFD) (Figure 3F).

### 3.4 Lipid metabolism and inflammatory factor gene expression

**FIGURE 4:**
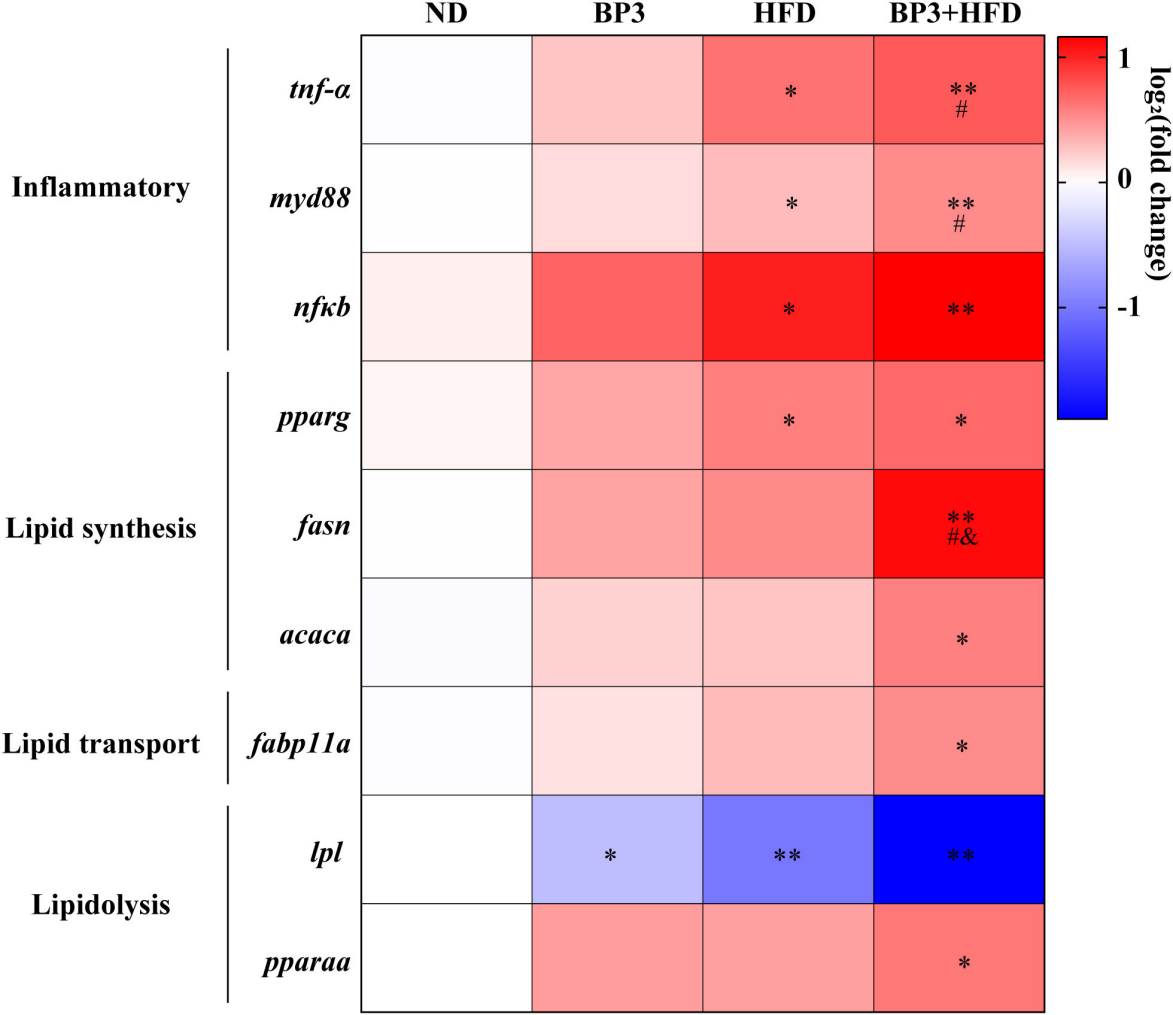
Heat map of gene transcription changes related to lipid metabolism and inflammatory factors in the liver of zebrafish exposed to BP3 under ND and HFD conditions. Red indicates upregulation, blue indicates downregulation. Color intensity represents the extent of changes. Values plotted are mean ± SEM. *P < 0.05 and **P < 0.01 indicate significant differences between the control and exposure groups. #*P* < 0.05 indicate significant differences when compared with BP3 group. &*P* < 0.05 indicate significant differences when compared with HFD group.

The genes *acaca* (*P* = 0.027 for BP3 + HFD) and *fsan* (*P* = 0.003 for BP3 + HFD), involved in lipid synthesis, showed increased expression levels across all treatment groups, with significant differences observed only in the BP3 + HFD group. Particularly, *fsan* expression was significantly higher in the BP3 + HFD group compared to both the BP3 (*P* = 0.036) and HFD (*P* = 0.049) groups. Similarly, the gene *fabp11a*, involved in lipid transport-regulating, exhibited a similar expression trend, albeit without statistical significance (*P* = 0.011 for BP3 + HFD). Conversely, the lipid catabolism-related gene *lpl* was significantly downregulated across all treatment groups (*P* = 0.047 for BP3, *P* = 0.004 for HFD, *P* = 0.002 for BP3 + HFD). Both members of the peroxisome proliferator-activated receptor (PPAR) family, *pparaa* (*P* = 0.012 for BP3 + HFD) and *pparg* (*P* = 0.038 for HFD, *P* = 0.011 for BP3 + HFD), showed marked upregulation in all treatment conditions. Furthermore, the pro-inflammatory mediators *nf*κ*b* (*P* = 0.014 for HFD, *P* = 0.002 for BP3 + HFD), *myd88* (*P* = 0.035 for HFD, *P* = 0.008 for BP3 + HFD), and *tnf-*α (*P* = 0.022 for HFD, *P* = 0.007 for BP3 + HFD) displayed varying levels of increased expression across the treatment groups. Notably, the BP3 + HFD group exhibited significantly higher levels of *tnf-*α (*P* = 0.044) and *myd88* (*P* = 0.044) compared to the BP3 group (Figure 4).

### 3.5 Histological alterations in zebrafish intestinal structure

**FIGURE 5:**
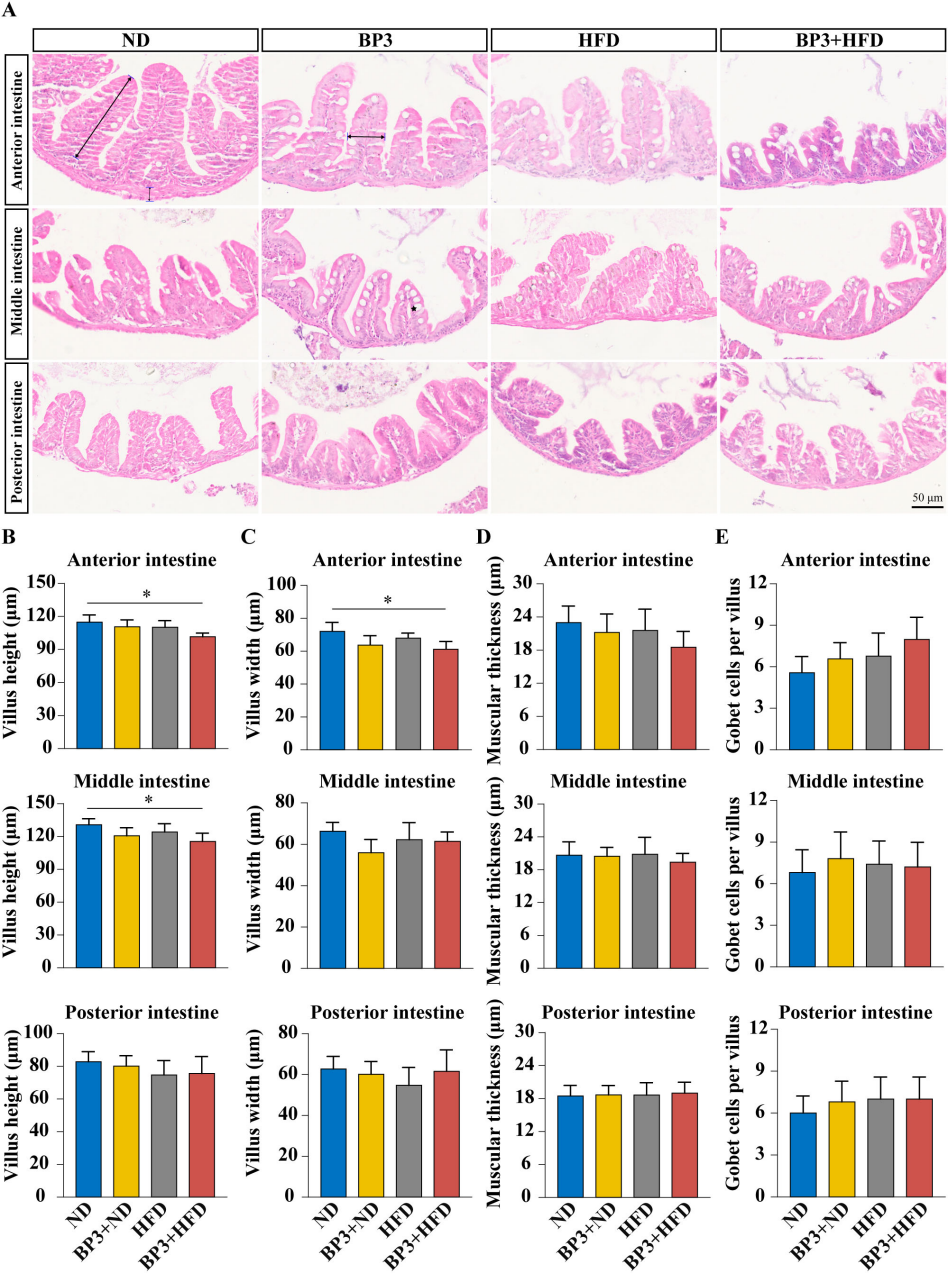
Effects of BP3 exposure under ND and HFD conditions on intestinal histopathology in zebrafish. **(A)** H&E staining (magnification at 400× and scale bar: 50 μm) of representative intestinal sections belonging to different groups, black pentagram represents goblet cells. **(B)** Intestinal villus height in different intestinal segments. **(C)** Intestinal villus width of different intestinal segments. **(D)** Intestinal muscular thickness of different intestinal segments. **(E)** Number of intestinal goblet cells in different intestinal segments. Values plotted are mean ± SEM. *P < 0.05 indicate significant differences between the control and exposure groups.

H&E staining revealed intestinal tissue damage across all treatment groups (Figure 5A). Villus height decreased in the intestine, with the BP3 + HFD group showing significant differences compared to the ND group in the anterior intestine (*P* = 0.028) and middle intestine (*P* = 0.031) (Figure 5B). Villus width followed a similar trend, with a significant reduction observed only in the anterior intestine of the BP3 + HFD group (*P* = 0.015) (Figure 5C). The muscular thickness showed a tendency toward thinning in all intestines (Figure 5D), while goblet cell numbers displayed an increasing trend across the intestine (Figure 5E). However, none of these parameters reached statistical significance.

### 3.6 Intestinal oxidative damage and serum LPS levels

**FIGURE 6:**
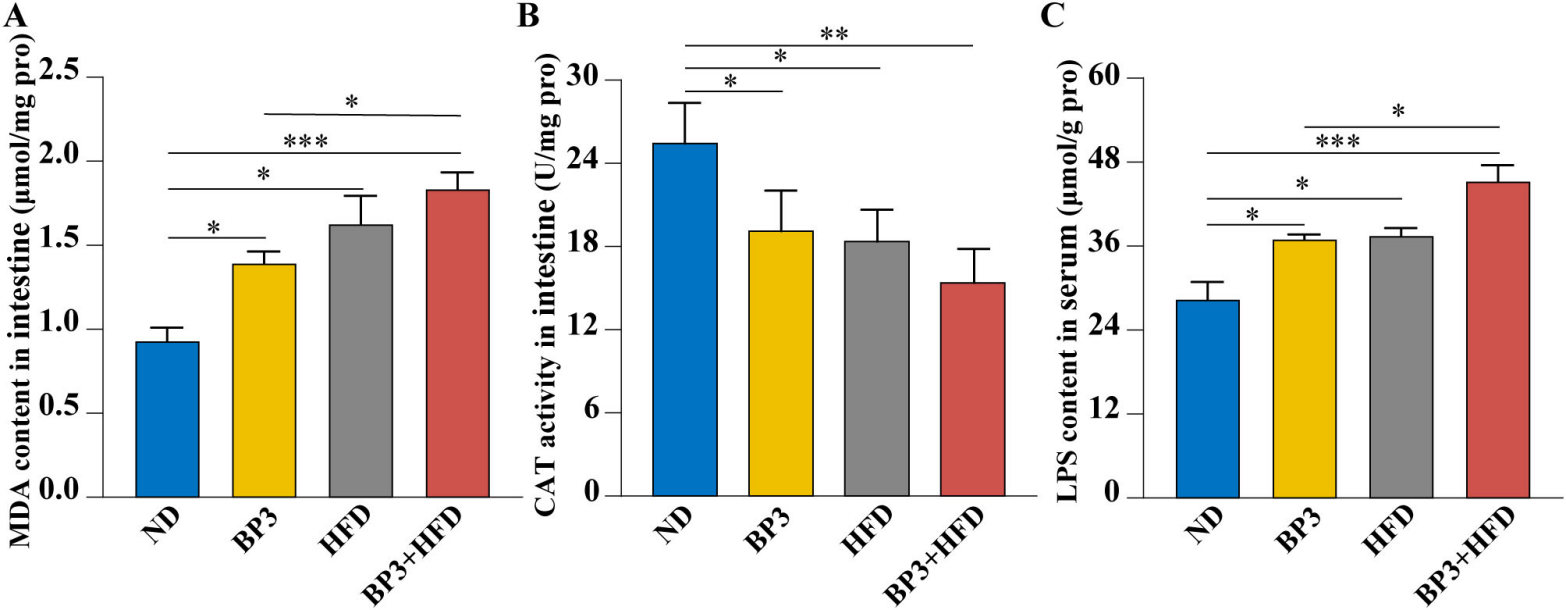
Effects of BP3 exposure under ND and HFD conditions on intestinal oxidative stress and serum LPS levels in zebrafish. **(A)** MDA contents and **(B)** CAT contents in intestine. **(C)** LPS contents in serum. Values plotted are mean ± SEM. *P < 0.05, **P < 0.01, ***P < 0.001 indicate significant differences between the control and exposure groups.

Intestinal oxidative damage markers showed notable dysregulation analogous to hepatic manifestations. The lipid peroxidation marker MDA was notably elevated (*P* = 0.033 for BP3, *P* = 0.004 for HFD, *P* < 0.001 for BP3 + HFD), while antioxidant enzyme CAT was significantly reduced (*P* = 0.041 for BP3, *P* = 0.014 for HFD, *P* = 0.001 for BP3 + HFD) across all treatment groups compared to the ND group. Furthermore, MDA levels were significantly higher in the BP3 + HFD group than in the BP3 group (*P* = 0.047) (Figures 6A, B). Notably, serum levels of intestinal-derived LPS were significantly increased across all treatment groups compared to the ND group (*P* = 0.024 for BP3, *P* = 0.020 for HFD, *P* = 0.002 for BP3 + HFD), and the BP3 + HFD group showed significantly higher levels than the BP3 group (*P* = 0.049) (Figure 6C).

### 3.7 Gut microbial alterations

**FIGURE 7:**
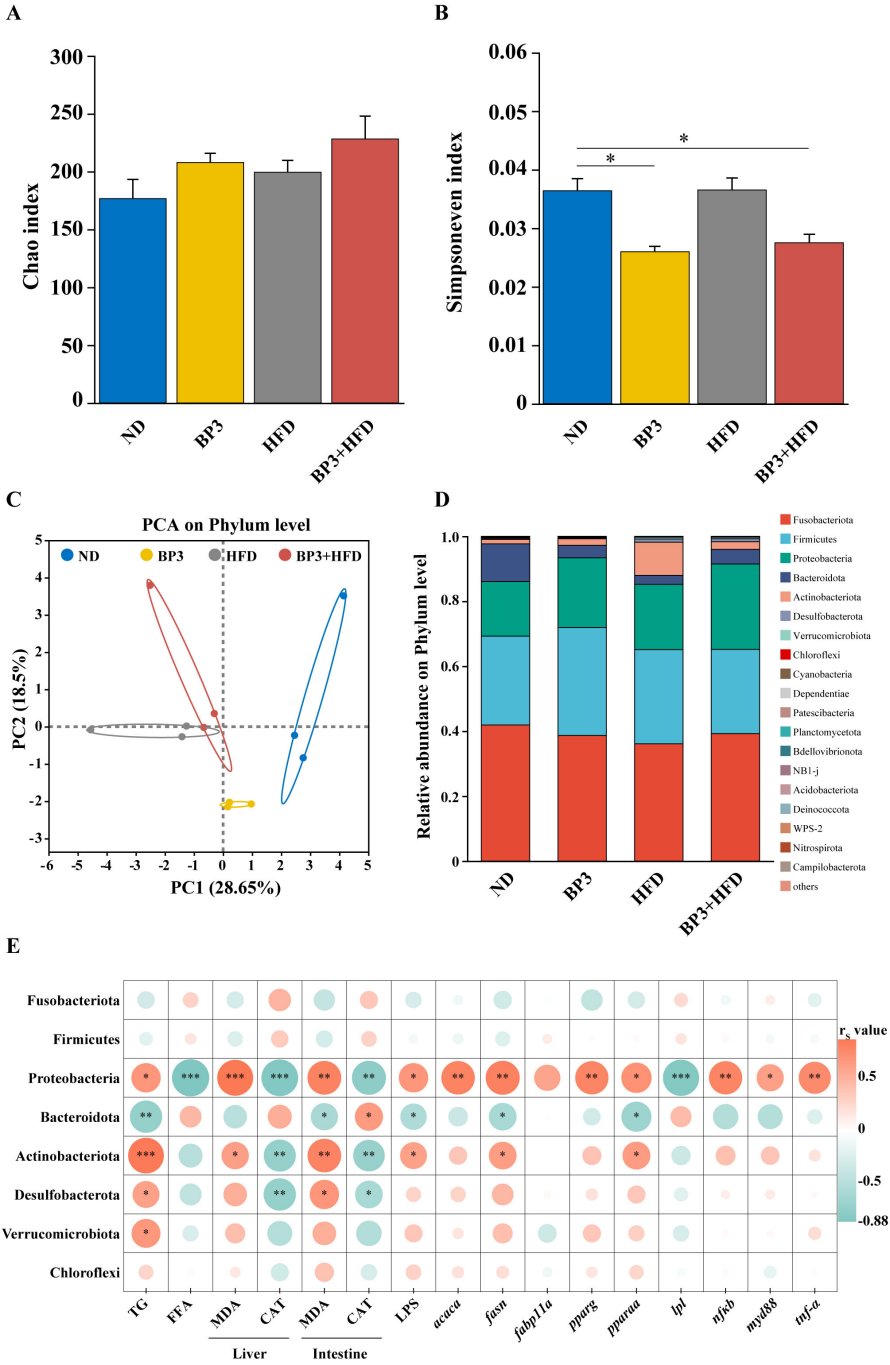
Alterations in gut microbiota composition at the phylum level in zebrafish following BP3 exposure under ND and HFD conditions. Alpha diversity analysis: **(A)** Chao index, **(B)** Simpsoneven index. **(C)** Beta diversity analysis. **(D)** Changes in relative abundance of gut microbiota at the phylum level. **(E)** Spearman correlation between detected index and abundance of top eight gut microbiota at the phylum levels. Red indicates positive correlation; green indicates negative correlation. Color intensity indicates the degree of correlation. Values plotted are mean ± SEM. *P < 0.05, **P < 0.01, ***P < 0.001 indicate significant differences between the control and exposure groups.

Experiment groups exhibited distinct gut dysbiosis compared to the ND group. Alpha diversity analysis revealed increased Chao indices alongside significantly reduced Simpson evenness indices in BP3 (*P* = 0.010) and BP3 + HFD (*P* = 0.024) groups, indicating specific impacts of chemical exposure on microbial community evenness. Principal component analysis (PCA, R^2^ = 0.89, *P* = 0.001) demonstrated clear separation of microbiota structures across groups (Figures 7A–C).

At the phylum level, five dominant taxa were identified: *Fusobacteriota* (ND: 0.42, BP3: 0.39, HFD: 0.36, BP3 + HFD: 0.39), *Firmicutes* (ND: 0.27, BP3: 0.33, HFD: 0.29, BP3 + HFD: 0.26), *Proteobacteria* (ND: 0.17, BP3: 0.22, HFD: 0.20, BP3 + HFD: 0.26), *Bacteroidota* (ND: 0.12, BP3: 0.04, HFD: 0.03, BP3 + HFD: 0.04), and *Actinobacteriota* (ND: 0.01, BP3: 0.02, HFD: 0.10, BP3 + HFD: 0.02). Exposure groups displayed marked phylum-level restructuring, characterized by decreased relative abundances of *Fusobacteriota* and *Bacteroidota*, contrasted by elevated proportions of *Proteobacteria* and *Actinobacteriota*. Notably, the *Firmicutes*/*Bacteroidota* (F/B) ratio showed a significant increase in experiment groups (Figure 7D).

To further elucidate the intrinsic relationships among oxidative stress, lipid metabolism, and alterations in gut microbiota composition, Spearman’s correlation analysis was employed to systematically evaluate the associations between oxidative stress indices, lipid metabolism parameters, and the top eight dominant bacterial phyla at the taxonomic level of phylum. Positive correlations were observed between *Proteobacteria* and lipid biosynthesis, oxidative indicators, and pro-inflammatory factors, while negative correlations were identified with lipid metabolism and antioxidant indicators. A similar trend was noted for *Actinobacteriota*. However, *Bacteroidota* displayed an inverse trend (Figure 7E). Specific correlation coefficients (r_*s*_ value) and corresponding *p*-value are provided in the Supplementary Tables 2, 3.

**FIGURE 8:**
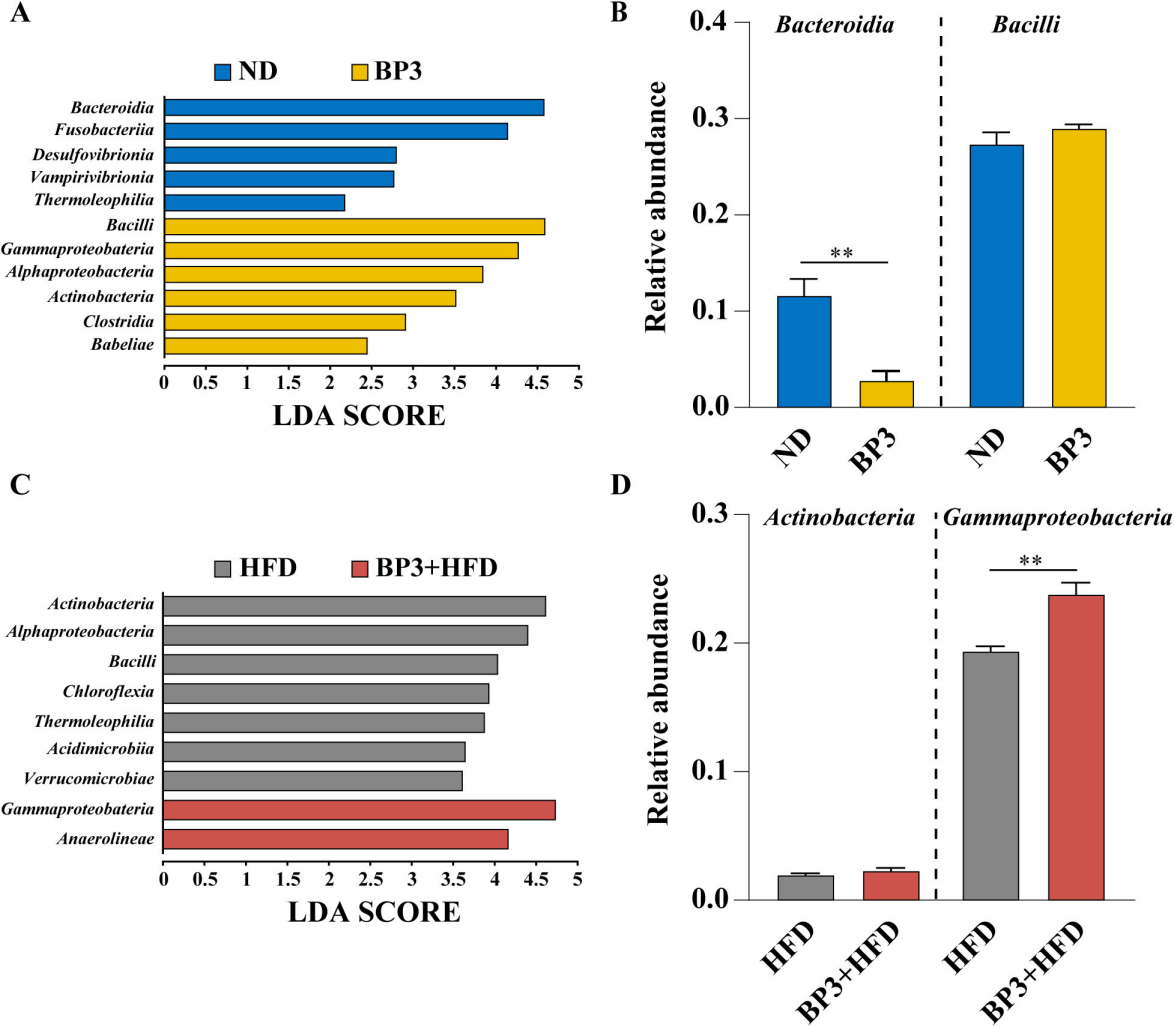
Linear Discriminant Analysis Effect Size (LEfSe) analysis identified diet-dependent alterations in sensitive intestinal taxa of zebrafish. **(A)** The most differentially abundant taxa between ND group and BP3 group. **(B)** Variation in the relative abundance of *Bacteroidia* and *Bacilli*. **(C)** The most differentially abundant taxa between HFD group and BP3 + HFD group. **(D)** Variation in the relative abundance of *Actinobacteria* and *Gammaproteobacteria*. Values plotted are mean ± SEM. **P < 0.01 indicates significant differences between the groups connected by the lines.

Linear discriminant analysis Effect Size (LEfSe) analysis demonstrated that zebrafish gut microbiota exhibited distinct class-level sensitivities under different diets: *Bacteroidia* (LDA = 4.58, *P* = 0.049) and *Bacilli* (LDA = 4.59, *P* = 0.049) were the most responsive taxa under normal diet (ND) (Figure 8A). *Actinobacteria* (LDA = 4.62, *P* = 0.049) and *Gammaproteobacteria* (LDA = 4.74, *P* = 0.049) dominated in HFD (Figure 8C). Further analysis of relative abundance revealed that *Bacteroidia* was significantly reduced in the BP3 group compared to the ND group (*P* = 0.002) (Figure 8B), while *Gammaproteobacteria* showed a marked increase in the BP3 with HFD group compared to the HFD group (*P* = 0.002) (Figure 8D).

## 4 Discussion and conclusion

This study constructed a zebrafish co-exposure model, which provides evidence of a significant interactive effect between the UV filter BP3 and high-fat diet (BP3 + HFD group) on exacerbating hepatic lipid deposition in NAFLD. The combination of BP3 and HFD exhibited more pronounced hepatic lipid accumulation, characterized by the presence of large lipid droplets and elevated TG levels. The primary synergistic mechanism is hypothesized to involve the breakdown of the gut-liver axis cascade in association with gut microbiota remodeling: co-exposure was association with specific dysbiosis, which was subsequently linked to damage of the intestinal barrier, potentially culminating in the systemic translocation of endotoxin LPS and the activation of the hepatic inflammatory signaling.

Zebrafish exposed to a combination of BP3 and HFD exhibited exacerbated characteristics of NAFLD compared to individual exposures of BP3 or HFD. This was evidenced by increased accumulation of larger lipid droplets in hepatocytes, TG levels, and upregulation of inflammation-related genes (*myd88*, *nfkb*, *tnf-a*) in the BP3 + HFD group. Furthermore, the co-exposed group showed heightened expression of key transcription factors involved in adipocyte differentiation and lipid synthesis (*pparg*), genes related to fatty acid synthesis (*acaca*, *fasn*), fatty acid transport (*fabp1*), and lipoprotein lipase (*lpl*) in their livers, exceeding levels observed in single exposure group. These findings indicate that under normal dietary conditions, BP3 induces subtle NAFLD phenotypes. However, exposure to a high-fat diet significantly exacerbates the effects of BP3, leading to worsened lipid degeneration in zebrafish livers compared to the HFD group or the BP3 group under normal diet conditions. Additionally, the BP3 + HFD group showed significantly increased MDA levels and decreased CAT activity in their livers, indicating increased oxidative damage. The above synergistic model aligns with previous study showing microplastics (MPs) enhancing oxytetracycline (OTC)-induced NAFLD symptoms (Zhou et al., 2023). These finding imply that certain environmental pollutants (e.g., BP3, MPs) under specific metabolic stressors (e.g., HFD, or in combination with another pollutant) can significantly trigger or amplify their hepatotoxic potential. This highlights the importance of considering their interaction with common dietary factors (e.g., high-fat diet) when evaluating the health risks associated with environmental pollutants.

The gut-liver axis, a bidirectional regulatory system connecting intestinal and hepatic functions, depends heavily on gut microbiota for functional coordination (Deng et al., 2023; Li et al., 2023; Liao et al., 2023). Disruptions in this microbial community can severely compromise gut-liver axis communication, particularly by interfering with intestinal barrier integrity (Hersoug et al., 2016). Our study revealed that co-exposure induced gut microbiota dysbiosis, manifested by a significant reduction in *Bacteroidota* and *Fusobacteriota*, alongside increased proportions of *Proteobacteria* and *Actinobacteriota*. This transition was further marked by an elevated *Firmicutes*-to-*Bacteroidota* (F/B) ratio, a key indicator of gut ecological imbalance strongly correlated with obesity-related metabolic disturbances (Ley et al., 2005). This dysbiosis was associated with intestinal barrier impairment, which likely occurred through two synergistic mechanisms: structural impairment, as evidenced by villus shortening in critical sites for absorption and barrier function in the anterior and middle intestine, and oxidative damage, indicated by elevated MDA levels and reduced CAT activity in the zebrafish intestine. Notably, goblet cells, vital for the intestinal barrier integrity (Bhatia et al., 2015), remained unaffected in our investigation, possibly due to the susceptibility of enterocytes at the villi apex to oxidative damage and toxicity. Nevertheless, further investigation is required to assess the compensatory mechanisms associated with mucus secretion quality, quantity, and functionality by goblet cells. The compromised intestinal barrier may have facilitated the systemic translocation of endotoxins (Martín-Mateos and Albillos, 2021), as evidenced by increased plasma LPS levels. Subsequently, translocated LPS may activate the hepatic *myd88*/*nf-*κ*b* signaling, leading to the secretion of pro-inflammatory *tnf-*α, thereby worsening NAFLD progression. This highlights the critical role of intestinal integrity in NAFLD development, as dietary factors and pollutants collectively disrupt gut dysbiosis. This dysbiosis, in turn, collaboratively compromises the intestinal barrier through villus damage and oxidative damage, facilitating endotoxin translocation and subsequent hepatic inflammation along the “dysbiosis-barrier disruption-endotoxin translocation” axis.

In this study, we also analyzed the correlations between the predominant gut phyla and host metabolic parameters. Our finding revealed significant correlations between variations in *Proteobacteria*, *Actinobacteriota*, and *Desulfobacterota* level and lipid accumulation, oxidative stress, and inflammatory responses, alongside inverse associations with lipid catabolism pathways. Our study further utilized LEfSe analysis to explore the effects of BP3 exposure on gut microbiota under different dietary conditions. The results revealed that *Bacteroidia* exhibited heightened sensitivity to BP3 in a normal diet environment, suggesting a potential targeted biological effect on this group. Conversely, under high-fat dietary conditions, *Gammaproteobacteria* were identified as the primary responder to BP3, indicating a shift in BP3’s influence toward this bacterial group. The decrease in *Bacteroidia* could potentially affect the generation of SCFAs and compromise intestinal barrier function. On the other hand, the rise in *Gammaproteobacteria*, commonly linked to intestinal inflammation and endotoxin release, may exacerbate NAFLD through the LPS-*myd88/nfkb* pathway (Di Vincenzo et al., 2024; Nogal et al., 2021), in line with the results presented above. These findings suggest that, in the zebrafish model, BP3 may indirectly contribute to metabolic disorders through the “microbiota-gut-liver” axis with pronounced adverse effects observed in high-fat dietary contexts.

This study provides evidence that the combined exposure to the UV filter BP3 and a high-fat diet exacerbates NAFLD-like phenotypes in zebrafish, potentially through disruption of the “microbiota-gut-liver” axis. This disruption results in dysbiosis, characterized by a *Firmicutes*/*Bacteroidetes* imbalance, as well as intestinal barrier impairment, including villus atrophy and intestinal oxidative stress. These changes may lead to LPS translocation, ultimately activating the liver *myd88*/*nf-*κ*b* pathway and inducing liver inflammation. The study presents a novel finding regarding BP3’s specific effect on diverse microbiota under different dietary conditions, highlighting a diet-dependent targeting effect on the microbiota. This provides a complementary perspective to the traditional approach of assessing pollutant toxicity independently and emphasizes the importance of the host’s metabolic status, particularly diet, as a potential critical factor in pollutant-microbiota interactions. This perspective offers new insights into comprehending the influence of environmental factors on metabolic diseases. Future assessments of environmental health risks and strategies for preventing NAFLD should take into account the host’s metabolic background.

## Data Availability

The data presented in this study have been deposited in the NCBI repository under Sequence Read Archive (SRA) accession number SRR35776795, BioProject accession number PRJNA1344653, and BioSample accession number SAMN52650265.

## Footnotes

1
http://github.com/OpenGene/fastp

2
http://www.mothur.org/wiki/Calculators

3
http://huttenhower.sph.harvard.edu/galaxy/

4
https://www.majorbio.com/
