# Supplementary material for: Benzophenone-3 remodels gut microbiota diet-dependently to exacerbate non-alcoholic fatty liver disease in zebrafish
Source: Front Microbiol. 2025 Oct 24;16:1694753. doi: 10.3389/fmicb.2025.1694753 (PMC12592189; doi:10.3389/fmicb.2025.1694753)
Supplement: Supplementary file 1 [file Supplementary_file_1.docx]

SUPPORTING INFORMATION


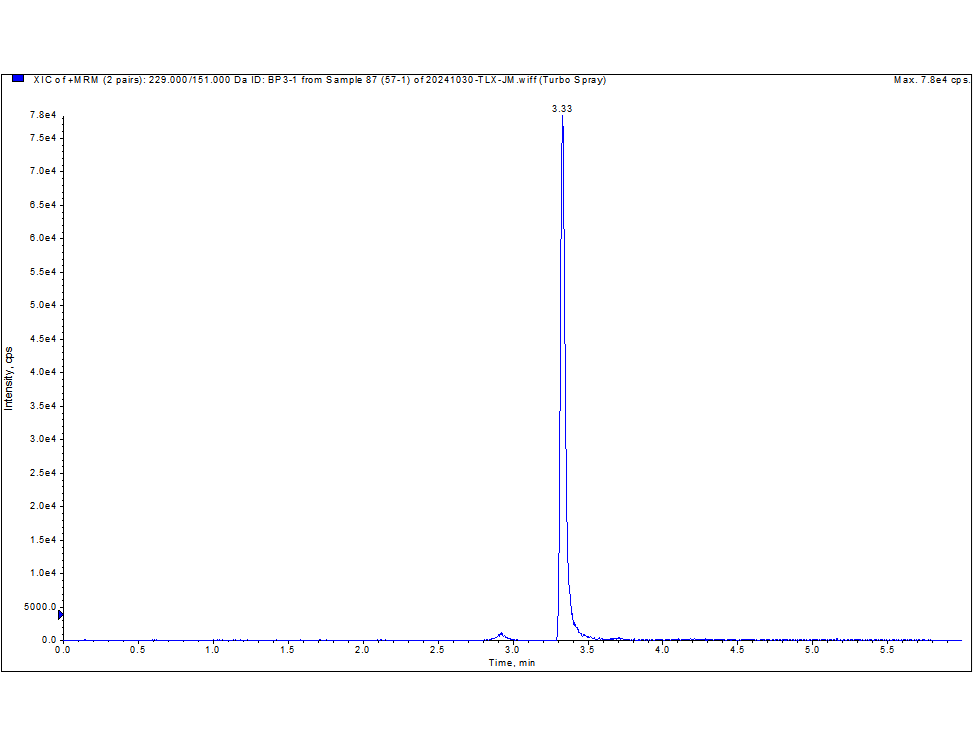


Fig. S1 Typical chromatography of BP3

Table. S1 Primer sequence for RT-qPCR

| Gene bank number | Gene | Forward primer | Reverse primer | Length (bp) |
| --- | --- | --- | --- | --- |
| XM_017356114.3 | *acaca* | GGACGGACCCTTGCACAATA | CCTCTGCAGGTCGATACGTC | 91 |
| XM_009306807.4 | *fasn* | ATGGAGTTTTCAGGGCGAG | GGGAATAATATGCGGTGGC | 175 |
| NM_001004682.1 | *fabp11a* | AGCACCTTCAAAACCACCGA | TCCCAGGTCTGTTTCTGCAC | 131 |
| XM_005164753.5 | *pparaa* | CTGGGAGACCCGATTA | TGCTGGCTGAGAACAC | 214 |
| NM_131467.1 | *pparg* | CTGCCGCATACACAAGAAGA | TCACGTCACTGGAGAACTCG | 152 |
| NM_131127.1 | *lpl* | GAATACACGGCGAGAAGGA | CAGTTTGCGAATGTGGAAGG | 188 |
| NM_001003414.1 | *nfκb* | AGAGAGCGCTTGCGTCCTT | TTGCCTTTGGTTTTTCGGTAA | 100 |
| NM_212814.2 | *myf88* | AACAACTTCGCTGGATAA | GTTACTGGAATCGCCTCA | 82 |
| NM_212859.2 | *tnf-α* | AGAAGGAGAGTTGCCTTTACCGCT | ACACCCTCCATACACCCGACTTT | 80 |
| NM_181601 | *β-αctin* | AAGCAGGAGTACGATGAGTC | TGGAGTCCTCAGATGCATTG | 238 |

| Table. S2 Spearman’s rank correlation coefficients (r_s_) | | | | | | | | |
| --- | --- | --- | --- | --- | --- | --- | --- | --- |
| r_s_ Value | Fusobacteriota | Firmicutes | Proteobacteria | Bacteroidota | Actinobacteriota | Desulfobacterota | Verrucomicrobiota | Chloroflexi |
| TG | -0.329 | -0.217 | 0.664 | -0.748 | 0.846 | 0.594 | 0.650 | 0.252 |
| FFA | 0.273 | 0.154 | -0.888 | 0.448 | -0.510 | -0.455 | -0.287 | -0.049 |
| MDA (liver) | -0.317 | -0.268 | 0.852 | -0.500 | 0.606 | 0.500 | 0.401 | 0.134 |
| CAT (liver) | 0.469 | 0.322 | -0.874 | 0.510 | -0.741 | -0.748 | -0.538 | -0.343 |
| MDA (intestine) | -0.435 | -0.316 | 0.789 | -0.604 | 0.786 | 0.663 | 0.502 | 0.375 |
| CAT (intestine) | 0.343 | 0.273 | -0.797 | 0.629 | -0.727 | -0.615 | -0.552 | -0.294 |
| LPS | -0.294 | -0.098 | 0.664 | -0.580 | 0.594 | 0.259 | 0.343 | 0.273 |
| *acaca* | -0.119 | -0.147 | 0.783 | -0.385 | 0.350 | 0.266 | 0.147 | 0.175 |
| *fasn* | -0.350 | -0.259 | 0.797 | -0.615 | 0.615 | 0.448 | 0.399 | 0.196 |
| *fabp11a* | -0.042 | 0.105 | 0.573 | -0.035 | 0.000 | 0.028 | -0.364 | -0.126 |
| *pparg* | -0.441 | 0.007 | 0.776 | -0.329 | 0.371 | 0.175 | 0.343 | 0.154 |
| *pparaa* | -0.322 | 0.021 | 0.692 | -0.685 | 0.622 | 0.343 | 0.287 | 0.245 |
| *lpl* | 0.224 | 0.154 | -0.860 | 0.427 | -0.378 | -0.231 | -0.294 | -0.070 |
| *nfkb* | -0.112 | 0.028 | 0.776 | -0.552 | 0.392 | 0.091 | 0.021 | 0.021 |
| *myd88* | 0.105 | -0.091 | 0.601 | -0.538 | 0.357 | 0.112 | 0.028 | -0.189 |
| *tnf-α* | -0.224 | -0.084 | 0.741 | -0.259 | 0.168 | 0.028 | 0.203 | -0.042 |

Table. S3 Spearman’s rank correlation *P* value

| *P* Value | Fusobacteriota | Firmicutes | Proteobacteria | Bacteroidota | Actinobacteriota | Desulfobacterota | Verrucomicrobiota | Chloroflexi |
| --- | --- | --- | --- | --- | --- | --- | --- | --- |
| TG | 0.297 | 0.499 | 0.018 | 0.005 | 0.001 | 0.042 | 0.022 | 0.430 |
| FFA | 0.391 | 0.633 | <0.001 | 0.145 | 0.090 | 0.138 | 0.366 | 0.880 |
| MDA (liver) | 0.316 | 0.400 | <0.001 | 0.098 | 0.037 | 0.098 | 0.196 | 0.678 |
| CAT (liver) | 0.124 | 0.308 | <0.001 | 0.090 | 0.006 | 0.005 | 0.071 | 0.276 |
| MDA (intestine) | 0.157 | 0.317 | 0.002 | 0.038 | 0.002 | 0.019 | 0.096 | 0.229 |
| CAT (intestine) | 0.276 | 0.391 | 0.002 | 0.028 | 0.007 | 0.033 | 0.063 | 0.354 |
| LPS | 0.354 | 0.762 | 0.018 | 0.048 | 0.042 | 0.417 | 0.276 | 0.391 |
| *acaca* | 0.713 | 0.649 | 0.003 | 0.217 | 0.265 | 0.404 | 0.649 | 0.587 |
| *fasn* | 0.265 | 0.417 | 0.002 | 0.033 | 0.033 | 0.145 | 0.199 | 0.542 |
| *fabp11a* | 0.897 | 0.746 | 0.051 | 0.914 | 1.000 | 0.931 | 0.245 | 0.697 |
| *pparg* | 0.152 | 0.983 | 0.003 | 0.297 | 0.236 | 0.587 | 0.276 | 0.633 |
| *pparaa* | 0.308 | 0.948 | 0.013 | 0.014 | 0.031 | 0.276 | 0.366 | 0.443 |
| *lpl* | 0.484 | 0.633 | <0.001 | 0.167 | 0.226 | 0.471 | 0.354 | 0.829 |
| *nfkb* | 0.729 | 0.931 | 0.003 | 0.063 | 0.208 | 0.779 | 0.948 | 0.948 |
| *myd88* | 0.746 | 0.779 | 0.039 | 0.071 | 0.255 | 0.729 | 0.931 | 0.557 |
| *tnf-α* | 0.484 | 0.795 | 0.006 | 0.417 | 0.602 | 0.931 | 0.527 | 0.897 |
